# Supplementary material for: Long-read based de novo assembly of low-complexity metagenome samples results in finished genomes and reveals insights into strain diversity and an active phage system
Source: BMC Microbiol. 2019 Jun 25;19:143. doi: 10.1186/s12866-019-1500-0 (PMC6593500; doi:10.1186/s12866-019-1500-0)
Supplement: Supplementary file 3 — The individual genome assemblies were deposited at NCBI Genbank with accession numbers. (DOCX 14 kb) [file 12866_2019_1500_MOESM3_ESM.docx]

| Sample | Genome(s) | BioProject | BioSample | Accession |
| --- | --- | --- | --- | --- |
| NWC_1 | *S. thermophilus* NWC_1_1; *Streptococcus* phage VS-2018a | PRJNA454439 | SUB3965239 | CP029252-CP029253 |
| NWC_1 | *L. delbrueckii* subsp. *lactis* NWC_1_2; pNWC_1_2 | PRJNA454439 | SUB3974946 | CP029250-CP029251 |
| NWC_2 | *S. thermophilus* NWC_2_1; *Streptococcus* phage ViSo-2018a | PRJNA477604 | SUB4196307 | CP031021-CP031022 |
| NWC_2 | *L. delbrueckii* subsp. *lactis* NWC_2_2;  pNWC_2_2; *Lactobacillus* phage ViSo-2018a; *Lactobacillus* phage ViSo-2018b | PRJNA477604 | SUB4196305 | CP031023-CP031026 |
| NWC_2 | *L. helveticus* NWC_2_3; pNWC_2_3 | PRJNA477604 | SUB4196309 | CP031016-CP031017 |
| NWC_2 | *L. helveticus* NWC_2_4; pNWC_2_3; pNWC_2_4 | PRJNA477604 | SUB4196317 | CP031018-CP031020 |
